# Intermediate uveitis associated with MS: Diagnosis, clinical features, pathogenic mechanisms, and recommendations for management

**DOI:** 10.1212/NXI.0000000000000909

**Published:** 2020-10-30

**Authors:** Alan Abraham, Lindsay Nicholson, Andrew Dick, Claire Rice, Denize Atan

**Affiliations:** From the Translational Health Sciences (L.N., A.D., C.R., D.A.), Bristol Medical School, University of Bristol; Bristol Eye Hospital (A.A.,A.D., D.A.), University Hospitals Bristol and Weston NHS Foundation Trust, United Kingdom; UCL- Institute of Ophthalmology and NIHR Biomedical Research Centre (A.D.), Moorfields Eye Hospital and UCL-Institute of Ophthalmology; and Clinical Neurosciences (C.R.), Southmead Hospital, North Bristol NHS Trust, United Kingdom.

## Abstract

Uveitis is a major cause of visual impairment and blindness among working-age adults, accounting for 10% of legal blindness in the United States. Among people with MS, the prevalence of uveitis is 10 times higher than among the general population, and because MS and uveitis share similar genetic risk factors and immunologic effector pathways, it is not clear whether uveitis is one of the manifestations of MS or a coincident disorder. This uncertainty raises several diagnostic and management issues for clinicians who look after these patients, particularly with regard to recognizing visual symptoms resulting from demyelination, intraocular inflammation, or the visual complications of disease modifying drugs for MS, e.g., fingolimod. Likewise, management decisions regarding patients with uveitis are influenced by the risk of precipitating or exacerbating episodes of demyelination, e.g., following anti–tumor necrosis factor biologic therapy, and other neurologic complications of immunosuppressive treatments for uveitis. In this review, we explore the similarities in the pathophysiology, clinical features, and treatment of patients with uveitis and MS. Based on the latest evidence, we make a set of recommendations to help guide neurologists and ophthalmologists to best manage patients affected by both conditions.

Uveitis is a major cause of visual impairment and blindness among working-age adults, accounting for 10% of legal blindness in the United States.^1^ Uveitis is traditionally defined as inflammation of the uveal tract, although inflammation is not confined to the uvea; consequently, uveitis is now defined anatomically based on the principal sites of inflammation: anterior uveitis affects the iris and ciliary body; intermediate uveitis (IU) predominantly affects the vitreous; posterior uveitis affects the retina and/or choroid; and panuveitis refers to anterior, intermediate, and posterior uveitis combined.^2^ The incidence of uveitis varies between 17.4 and 52.4 cases per 100,000 person years, and the prevalence between 69.0 to 114.5 per 100,000 persons,^3^ but among patients with MS, the prevalence is 1%.^4^

MS is an inflammatory demyelinating disease of the CNS, affecting almost 2.5 million people worldwide.^5^ It is frequently associated with visual symptoms caused by demyelinating lesions of afferent and efferent visual pathways. IU is the uveitis subtype most commonly associated with MS, but because retinal neurons are normally unmyelinated, IU is not a consequence of demyelination. Yet it is still not known whether IU is one of the manifestations of MS or a coincident disorder. This raises several diagnostic and management issues for clinicians who look after patients affected by both disorders with regard to recognizing visual symptoms resulting from demyelination, intraocular inflammation, or the complications of treatment.

This review summarizes the common pathophysiology and clinical features of IU and MS to draw inferences regarding the optimal management of patients affected by both conditions.

## Common pathways in the pathogenesis of MS and IU

The eye and brain are immune-privileged sites, created by tight junctions between vascular endothelial cells and the cytokine milieu. Inflammation occurs through breakdown of the normal immunoregulatory mechanisms in the eye and brain. Although it is still unclear what triggers inflammation in both conditions, several sources of evidence suggest that they share similar risk factors and immunologic effector pathways.^6,7^

### Common risk factors for MS and IU

Environmental risk factors, including exposure to Epstein-Barr virus, smoking, northern latitude, and low vitamin D are associated with MS,^7^ with evidence for an immunoregulatory role of the gut microbiome.^8^ These risk factors are not linked to IU, although the etiology of uveitis varies worldwide: 30%–50% of cases are caused by infection in developing nations, whereas a greater proportion are attributed to noninfectious, immune-mediated mechanisms in higher-income countries.^3^

**Figure 1:**
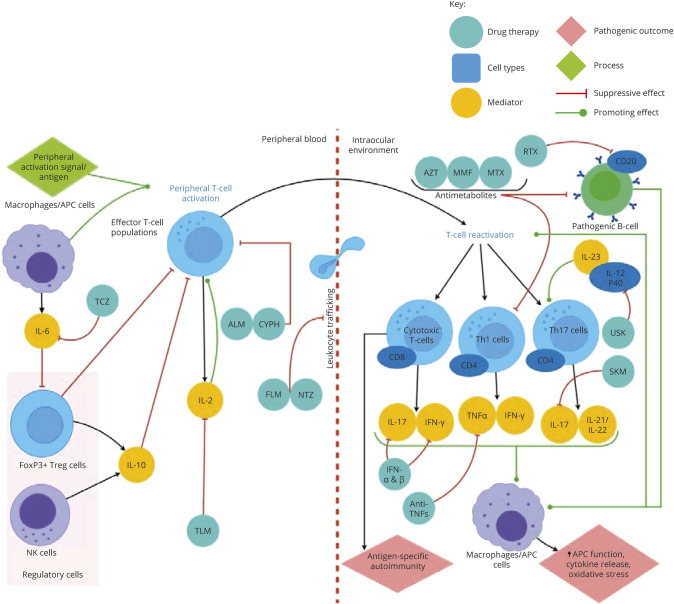
Model for uveitis immunopathogenesis and the effects of immunotherapies on its mediators Uveitis is considered to be a T cell–mediated disease driven by CD4^+^ Th1/Th17 cells. Release of major cytokines, IL-17 and IFN-γ, activates inflammatory cascades, which disrupt the blood-retinal barrier, and causes local tissue damage via reactive oxygen species (ROS), nitric oxide synthesis (NOS), and cell-mediated damage. Antigen-presenting cells (APCs) activate CD4 T cells and are further activated by their respective cytokines, which accentuate their function. Pathogenic B-cell populations are less well described, but also contribute to uveitis manifestations in humans via antigen specific autoimmunity and release of proinflammatory cytokines. Regulatory cells, including FoxP3^+^ Treg cells, suppress or control the manifestations of uveitis. Therapeutic agents with their proposed actions on key pathways in uveitis are highlighted in this figure. AZT = azathioprine; ALM = alemtuzumab; CYPH = cyclophosphamide; FLM = fingolimod; IFN = interferon; MMF = mycophenolate mofetil; MTX = methotrexate; NTZ = natalizumab; RTX = rituximab; SKM = secukinumab; TCZ = tocilizumab; TLM = tacrolimus; USK = ustekinumab.

**Figure 2:**
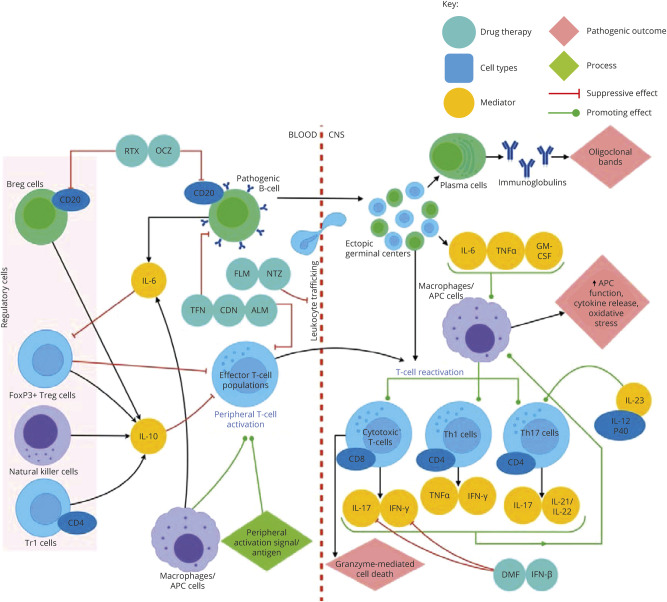
Model for MS immunopathogenesis and effects of immunotherapies on its mediators Episodes of acute demyelination in relapsing-remitting MS are mediated by T cells and B cells. The effector T cells are CD4^+^ Th1/Th17 cells, which release their respective lineage cytokines and promote further disease manifestations via their direct effects on CD8^+^ T cells and their indirect effects caused by cytokine release, for example, leading to the augmentation of APC function. They are activated in the periphery and reactivated in the CNS. Effector pathogenic B-cell populations form local lymphoid follicle–like collections within the CNS as disease becomes progressive, leading to the formation of plasma cells, which generate Igs and oligoclonal bands detected in the CNS. Innate immune cells such as APCs activate T-cell populations and are further activated by the cytokines released by CD4 T cells and pathogenic B cells. Natural killer cells have both pro- and anti-inflammatory roles in MS but form part of the regulatory milieu. Regulatory T-cell populations include the FoxP3^+^ Treg cells and CD4^+^ Tr1 cells, which produce anti-inflammatory cytokines and inhibit effector CD4^+^ T cells. Disease-modifying drugs for MS act by blocking leukocyte trafficking, e.g., FLM and NTZ, or they directly suppress pathogenic B cells and effector T-cell populations, e.g., TFN, CDN, and ALM, or they inhibit proinflammatory cytokines, e.g., IFN-β and DMF, or they suppress B cells by blocking CD20 activity, e.g., RTX and OCZ. ALM = alemtuzumab; APC = antigen-presenting cell; CDN = cladribine; DMF = dimethyl fumarate; FLM = fingolimod; IFN-β = interferon-beta; Ig = immunoglobulin; NTZ = natalizumab; OCZ = ocrelizumab; RTX = rituximab; TFN = teriflunomide.

The associations between MS and uveitis with infection support the hypothesis that they may be triggered by infectious agents in genetically susceptible individuals. Genome-wide association studies have identified loci accounting for up to 30% of an individual's risk of MS,^7^ and many overlap with genetic risk factors for IU, notably, human leukocyte antigen (HLA) class II genes, *HLA-DR15* and *HLA-DR-51.*^4^ Other shared genetic risk loci provide clues to immunologic effector pathways common to both disorders: tumor necrosis factor (*TNF*, rs361525, rs1800629), lymphotoxin alpha (rs909253), interleukin 6 (*IL-6*, rs1800795), IL-2/IL-21 (rs6822844), IL-2 receptor alpha (rs2104286, rs12722489), interferon regulatory factor 5 (rs10954213),^7,9,10^ and through one genetic linkage study, functional variants affecting TNF receptor superfamily members 10a and 13b (B cell–activating factor), G-protein subunit gamma transducing-1, alpha-2-macroglobulin domain containing-8, diacylglycerol kinase iota and reelin.^11^ Further support for their role in MS and uveitis pathogenesis comes from animal models (section Common immunologic effector mechanisms in MS and IU, figures 1 and 2).

### Common immunologic effector mechanisms in MS and IU

Experimental autoimmune uveitis (EAU) and experimental autoimmune encephalitis (EAE) are commonly used animal models of uveitis and MS, respectively, and there is evidence for cross-reactivity between the antigens used to precipitate them. Transient bilateral anterior uveitis occurs in EAE induced by myelin basic protein (MBP),^12^ whereas panuveitis occurs in EAE induced with S100B.^13^ MBP is also used to induce EAU.^14^ These experimental data imply some commonality in the precipitants of MS and uveitis that is further supported by evidence of autoreactive T cells from patients with MS displaying proliferative responses to retinal arrestin.^15^

In the eye (figure 1), the ocular microenvironment normally favors T-cell differentiation to the regulatory FoxP3^+^ (Treg) phenotype, maintaining ocular immune privilege.^16^ Likewise, FoxP3^+^ Tregs, Tr1 cells, and a subset of regulatory B cells (Bregs)^17,18^ limit immune activation in the brain (figure 2). However, compartmentalization of autoantigens in the eye and brain impairs the development of peripheral tolerance in autoreactive T cells, which can then precipitate uveitis and MS relapses. Evidence from EAU models suggests activated pathogenic T cells entering the eye fail to respond to regulatory cues and contribute to immune-mediated tissue damage via release of reactive oxygen species, nitric oxide synthesis, and cell-mediated damage.^16^ Progressive forms of MS are similarly thought to represent compartmentalized immune responses: B cells, microglia, and astrocytes may initiate the immune response, but trafficking of immune cells from the periphery becomes less important as disease progression becomes independent of these cells and progressive mitochondrial injury, oxidative stress, and ion channel redistribution ensue.^17,18^

Key effector cells in uveitis and MS relapses include distinct subsets of CD4^+^ T-helper 1 (Th1) cells producing signature cytokines interferon (INF)-γ and TNF-α and CD4^+^ T cells producing IL-17 (Th17).^6,19^ These cytokines activate an acute inflammatory cascade with recruitment of macrophages and neutrophils.^6^ In addition, CD8^+^ T cells and B cells are implicated: in postmortem specimens from patients with MS, CD8^+^ T cells are significantly enriched in perivascular cuffs and acute parenchymal lesions.^20^ Furthermore, 90% of patients with MS have oligoclonal bands in their CSF (intrathecally synthesized IgG),^21^ and lymph node–like follicles containing B cells have been identified adjacent to cortical MS lesions.^22^ Likewise, B-cell inflammatory infiltrates have been demonstrated in aqueous samples and chorioretinal biopsies from patients with active uveitis,^6,23^ and lymph node–like follicles can be found in the eyes of some patients with persistent uveitis.^24^ Hence, similar immune cell populations (CD4^+^ Th1 and Th17 cells, CD8^+^ cytotoxic T cells, Tregs, B cells, macrophages, and NK cells) and cytokines (TNF, IFNγ, IL-2, IL-6, IL-10, IL-12, IL-17, and IL-21/22/23) are involved in the pathogenesis of MS and uveitis (summarized in figures 1 and 2).

## Shared clinical features of MS and IU

### Diagnostic criteria

IU accounts for 10%–20% of uveitis cases overall, but 61%–80% of MS-associated uveitis.^3,11^ At present, MS-associated uveitis is not defined separately from undifferentiated (formerly idiopathic) IU, a term normally reserved for anatomically defined IU that is not associated with infection or systemic diseases, like sarcoidosis or Behçet disease.^2^ This is because a sizable proportion of people first diagnosed with IU might develop MS several years later.^4^ Neuroimaging is not currently recommended for patients with IU unless they already have neurologic symptoms or signs or they are being evaluated for certain biologic therapies (section Recommendations for management of MS-associated IU).^25^ Consequently, there are no known predictive clinical or investigation findings to identify those people with IU at greater risk of developing MS later. Because of the difficulties in defining MS-associated IU in patients who do not already have MS, the most conservative approach is to use the latest 2017 McDonald criteria for MS^5^ and the anatomic definition of undifferentiated IU by the Standardization of Uveitis Nomenclature Group.^2^

### Shared clinical symptoms and signs

The clinical presentation of IU differs from demyelinating lesions of the afferent and efferent visual pathways. Patients with IU may be asymptomatic for several years or develop symptoms insidiously. The most common symptoms (if they occur) are floaters, blurred vision, pain, photophobia, and red eye. Although symptoms of eye pain, blurred vision, and photophobia are also experienced by patients with acute optic neuritis (which affects 30%–50% of patients with MS),^26^ they normally start to improve spontaneously after a few weeks, although some degree of optic atrophy, reduced acuity, color vision, visual field, and contrast sensitivity may be long-term outcomes. In contrast, symptoms of IU tend to persist without treatment and are more likely to be confused with chronic optic neuropathy associated with progressive forms of MS.^26^ However, IU is not associated with an RAPD, unless it becomes complicated by optic disc edema (which is uncommon). Nor is undifferentiated IU associated with any neurologic symptoms. Examination findings include vitreous opacities (snowballs), exudates around the vitreous base or ora serrata (snowbanking), and peripheral periphlebitis, sometimes associated with vitreous hemorrhages.^27^

### Clinical course

**Figure 3:**
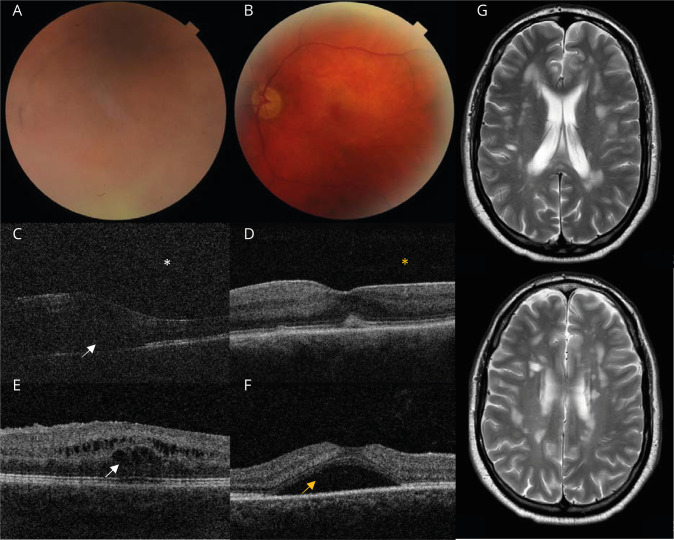
Fundal changes associated with intermediate uveitis and the complications of treatment Fundus photographs (A and B) and corresponding OCT images (C and D) taken from the same patient before (A and C) and after (B and D) treatment for IU. (A and C) Pretreatment images show how severe active vitritis makes the fundal view hazy (A) and blur OCT images of the retina (C). The white asterisk highlights the appearance of vitritis on OCT, which looks like a gray snowstorm. The white arrow points to coincident neuroretinitis causing retinal thickening in the same patient. (B and D) Posttreatment images show how the resolution of active vitritis mean that the fundal view (B) and OCT image of the retina (D) become clearer and the vitreous appears black (yellow asterisk). (E) OCT image showing cystoid macular edema: a complication of IU and fingolimod. The white arrow points to intraretinal cystic spaces and retinal thickening. (F) OCT image of central serous chorioretinopathy: a complication of steroid treatment. The yellow arrow points to subretinal fluid. (G) Two MRI brain slices from the same patient who developed demyelination following exposure to anti-TNF biologic therapy, demonstrating high T2 signal in the periventricular and deep white matter. Images are supplied courtesy of Dr. C. Rice, Dr. L. Kobayter, and Mr. T. Burke. IU = intermediate uveitis; OCT = optical coherence tomography.

Although IU typically affects both eyes, it usually has a good long-term visual prognosis. For example, the Multicenter Uveitis Steroid Trial found that patients with IU had a relatively good prognosis, except when macular thickening and edema were detected on optical coherence tomography (OCT) scans combined with active inflammation (figure 3C).^28^ Moreover, a retrospective case review at a tertiary center found that 22.5% of patients with IU did not require treatment, and 60% had relatively preserved visual acuity after 10 years of follow-up.^29^ Hence, the aim of management is to treat sight-threatening features (not uncomplicated or asymptomatic IU) such as glaucoma, cataract, epiretinal membrane, optic disc edema, retinal vasculitis, and retinal detachment. As these complications can develop insidiously, regular follow-up by an ophthalmologist is required to manage them.

Typically, the onset of MS-associated IU is in middle age. Patients with MS-associated IU are more commonly female with relapsing-remitting MS (RRMS); however, these features likely reflect the female preponderance and higher prevalence of RRMS overall. The prevalence of periphlebitis and other vasculitic changes in the retinal periphery is reportedly higher in patients with IU with MS compared with those without, although their clinical significance is uncertain because the visual prognosis of IU in patients with or without MS appears to be similar.^29^

It is not known whether IU is a predictor of worse MS disability as reports have been conflicting.^4,30^ A thinner retinal nerve fiber layer on OCT imaging is linked to worse MS disability,^31^ but because patients were not stratified by those with or without IU, the association is likely to reflect previous episodes of optic neuritis rather than IU. The relapse rate is higher in patients with MS with uveitis in clinical trials of fingolimod.^32^ Hence, it is possible that patients with MS-associated IU may have different MS prognostic or treatment outcomes, but the evidence in this area is still lacking.

## Shared treatment approaches in the management of MS and IU

Available treatments for MS and IU aim to reduce symptoms and cumulative visual or neurologic disability, but the complications of treatment can also affect visual and neurologic function (tables 1 and 2). The unintended consequences of treatment need careful differentiation from disease relapses or progression because their management will differ.^33^ The multidisciplinary management of patients by a neurologist and ophthalmologist is recommended for the best outcomes.

**Table 1:**
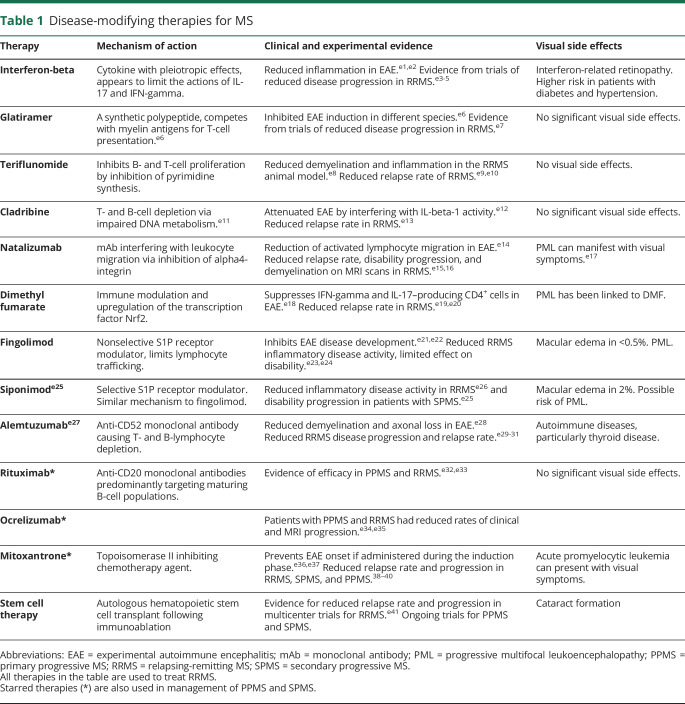
Disease-modifying therapies for MS

**Table 2:**
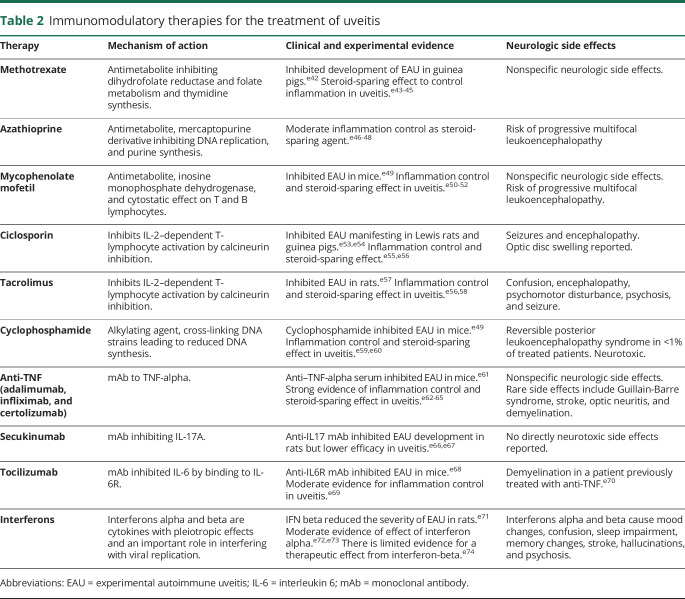
Immunomodulatory therapies for the treatment of uveitis

### Management of acute relapses of MS and IU

In the acute setting, MS relapses and sight-threatening inflammation in IU are both managed with corticosteroids. Oral or IV methylprednisolone will shorten the duration of an MS relapse, but does not have any meaningful impact on long-term neurodisability.^5^ Systemic steroids are used to treat acute relapses of IU; but unlike MS, intraocular inflammation is amenable to local therapy. Topical steroid drops do not penetrate the posterior segment of the eye, but periocular steroid injections or intravitreal injection of steroid implant are viable alternatives, which avoid the side effects of systemic corticosteroids. Moreover, the effects of intravitreal steroid implants can last up to 6 months.^34^

Important considerations are the possible complications of corticosteroid treatment and how they may be distinguished from relapses of MS or IU. Corticosteroids can cause systemic symptoms that may be confused with progressive neurodisability from MS. Likewise, frequent or chronic treatment with local or systemic corticosteroids can cause sight-threatening complications, like cataract, glaucoma and, central serous chorioretinopathy (CSCR).

Cataracts cause progressive reduction in visual acuity, contrast sensitivity, night vision, and color perception, which might be confused with optic neuropathy. Nevertheless, cataracts are not a contraindication to steroid treatment, and cataract surgery will restore vision. Glaucoma also causes insidious visual loss that is largely asymptomatic until advanced. It is a progressive optic neuropathy that is distinguished from demyelinating optic neuropathy based on characteristic optic nerve cupping. However, glaucoma can be treated medically and/or surgically to prevent permanent visual loss and represents a relative contraindication to local steroid treatment.^33^ Importantly, cataracts and glaucoma are also common sight-threatening complications of IU.

CSCR is another complication of local/systemic steroid treatment. The population incidence of CSCR is 9.9 per 100,000 in men and 1.7 per 100,000 in women, but among patients on long-term steroids, the prevalence is as high as 1%–6%.^35^ CSCR causes symptoms of visual distortion and central visual loss, and OCT imaging shows subretinal fluid in the central macular region (figure 3F). The condition is generally reversible following steroid withdrawal, but represents a relative contraindication to steroid treatment because chronic CSCR can lead to permanent visual loss.^35^ Without OCT imaging, the condition may be confused with macular edema associated with active inflammation in IU or the side effect of certain disease modifying drugs (DMDs) for MS, e.g., fingolimod (figure 3E, section Immunomodulatory drugs for MS and IU).

### Immunomodulatory drugs for MS and IU

Patients with MS with frequent relapses and patients with IU with sight-threatening or steroid-resistant disease may require additional immunomodulatory therapies. Several DMDs are now available to reduce the frequency of relapses in RRMS, and recent trials have shown promise for ocrelizumab and siponimod in primary and secondary progressive MS^18,36,37^(summarized in figure 2 and table 1). Similarly, a range of immunomodulatory treatments for the whole spectrum of uveitic disorders is available for inflammation refractory to local or systemic steroids (summarized in table 2 and figure 1).^38^ As large clinical trials of medical treatments for uveitis normally include heterogeneous groups of patients with different uveitis subtypes, the choice of medical treatment for IU specifically is more difficult. Expert consensus statements are available, albeit not specific to IU.^38^ Most first-line immunosuppressants used to treat uveitis act by suppressing T- and B-cell activation and/or proliferation. Anti-TNF biologic therapies are usually reserved for patients with uveitis with disease refractory to first-line immunosuppressants, but there is a risk of precipitating demyelination (see below).^38^ The case for first-line anti-TNF treatment is stronger in patients with uveitis with other systemic diseases like Behçet disease.

Few studies have specifically examined the impact of established treatments for IU on MS or MS on IU, but as many immunomodulatory treatments for MS and uveitis target the same effector cells and/or leukocyte trafficking from the blood to the CNS, there is great potential for these agents in treating patients with coexistent disease. IFN-α is an effective treatment for uveitis, particularly associated with Behçet disease, but there is weaker evidence for the efficacy of IFN-β in uveitis.^38^ Isolated case reports and retrospective studies have reported improvements in patients with IU coincidentally started on glatiramer acetate or mycophenolate mofetil (MMF) for the management of MS, although evidence for MMF is much stronger in uveitis than MS. Azathioprine has been used to treat both MS and uveitis, but the evidence base is weaker.^38–40^ Studies of EAU support the use of fingolimod during active uveitis,^41^ but as human clinical trial data are currently lacking, one must weigh the benefits against the risk of ocular complications (see below). Furthermore, the relapse rate is higher in patients with MS with uveitis in clinical trials of fingolimod.^32^ More recently, anti–IL-6 receptor monoclonal antibodies (tocilizumab) and anti–IL-17 therapy (secukinumab) have been considered as additional options for treatment-refractory uveitis.^42^ Tocilizumab reduces inflammation in EAE and has been used to treat patients with neuromyelitis optica, suggesting that tocilizumab might be a good option in the treatment of both conditions.^43,44^ In 1 patient with tumefactive MS, treatment with natalizumab led to near-complete resolution of coincident IU,^45^ but there are no clinical trials supporting the use of natalizumab in IU. Alemtuzumab, which inhibits the activation of effector T cells in the peripheral circulation, has been reported to improve treatment-refractory uveitis.^46^ Furthermore, anti-CD20 therapies have been shown to be independently effective in uveitis and MS.^18,47^

The main limitation for using DMDs designed for MS to treat patients with uveitis is the risk of causing visual complications. These complications need to be distinguished from demyelinating optic neuropathy and IU (table 1). Macular edema associated with S1P inhibitors is the most significant: approximately 0.2% of patients on fingolimod develop macular edema within the first 6 months of treatment, and the incidence may be higher in patients with diabetes mellitus. Baseline OCT imaging is recommended before initiation of fingolimod treatment, with a second evaluation at 3–4 months.^48^ Symptoms of fingolimod-induced macular edema are identical to macular edema associated with IU and similar to CSCR but resolve following the withdrawal of fingolimod. Hence, OCT imaging is indicated in patients with MS who develop visual distortion or central visual loss to differentiate between the complications of IU, DMDs like fingolimod, and corticosteroid treatment (figure 3).

Similarly, all of the immunomodulatory treatments used to treat IU can cause neurologic side effects, and many of these could be confused with the onset of MS symptoms (table 2). Nonspecific neurologic symptoms such as confusion, dizziness, paresthesia, and muscle weakness are common. Furthermore, complications arise because of increased immunosuppression and the associated risks of JC viral infection and neoplasia.

An additional concern among ophthalmologists is the risk of precipitating new-onset demyelination and MS following treatment with biologic therapies. Experimental evidence that anti-TNF agents were effective in EAE led to clinical trials that paradoxically showed anti-TNF agents precipitated and exacerbated demyelination in patients with MS (figure 3G).^49^ There are also reports of CNS demyelination in patients with rheumatoid arthritis treated with tocilizumab.^42,50^ Why anti-TNF and anti–IL-6 receptor agents might precipitate demyelination in people not known to have MS or exacerbate demyelination in those who do is unclear, but underline the caveats of evidence derived from animal models. Consequently, ongoing caution and surveillance are required when using biological therapies to treat uveitis.

### Recommendations for management of MS-associated IU

There are several challenges for clinicians managing patients with MS and IU. First, it is difficult to detect uncomplicated IU in patients with MS without ophthalmic equipment or expertise. Second, the complications of chronic intraocular inflammation, e.g., cataract, glaucoma, and macular edema, are also complications of treatments for MS and IU, e.g., corticosteroids and fingolimod. In addition, several DMDs for MS cause visual side effects that could be confused with episodes of demyelination and the complications of IU or its treatment. Likewise, treatments for uveitis can cause neurologic side effects and demyelination. Multidisciplinary team working between ophthalmologists and neurologists is, therefore, key to ensuring better treatment outcomes for patients with MS and IU.

In patients with MS who are naive to DMDs, the development of IU is not an indication to start DMDs; management should be tailored to that required for uveitis. In patients with MS already on DMDs, local therapies for IU such as intravitreal dexamethasone implants are likely to work best to treat local inflammation while avoiding additional systemic side effects from escalating immunomodulatory treatment. Decisions regarding changes to DMDs for MS would also benefit from ophthalmology input to optimize the management of both conditions.

For patients with IU treated with anti-TNF agents who later develop demyelination, it is not clear whether these demyelinating episodes would have occurred in these patients regardless of whether they were treated with anti-TNF agents or not. This is because it is still not known whether IU is an early manifestation of MS or whether demyelination is purely a complication of anti-TNF treatment in those who would never have developed demyelination otherwise. Our recommendation is that all patients with IU have a brain MRI scan to determine their risk of developing clinically relevant demyelination before the introduction of anti-TNF therapy. For those patients with white matter lesions suggestive of demyelination (figure 3G), we consider anti-TNF therapy to be contraindicated pending further neurologic assessment, and all other treatment options for uveitis should be explored instead.

In patients with coexistent MS and IU, based on our current understanding of their pathophysiology, informed by disease models, case reports, and clinical trials, it is possible to make tentative recommendations for treatments that target specific aspects of the immune response common to both conditions. There is evidence pointing to the dual efficacy of IFNβ, glatiramer acetate, MMF, natalizumab, alemtuzumab, and anti-CD20 therapies in the treatment of both MS and IU, suggesting that treatments, which target leukocyte trafficking, B cells, or effector T cells in the peripheral circulation, may be most effective (table 3). However, there is a need for additional clinical trials in this area.

**Table 3:**
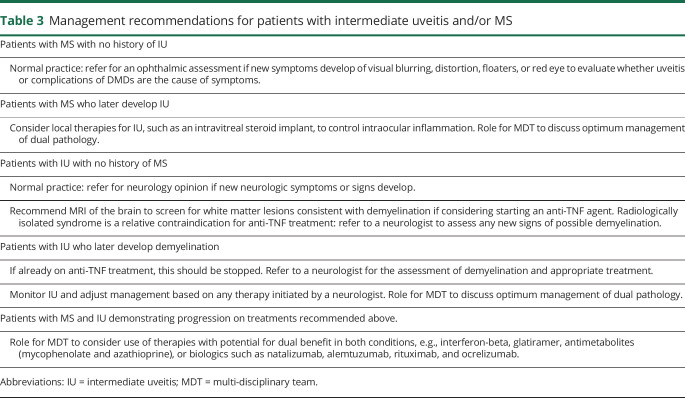
Management recommendations for patients with intermediate uveitis and/or MS

## Conclusions

MS and IU are both immune-mediated inflammatory diseases affecting immune-privileged sites in the eye and brain. Both disorders share similar immunopathogenic mechanisms, and, consequently, many of the same treatments are effective in the treatment of acute relapses and chronic inflammation in MS and IU, with the clear exception of anti-TNF therapies. An important consideration is that many of the treatments for MS and IU can cause visual and neurologic side effects and complications that may be confused with progression of either disease without careful examination, imaging and multidisciplinary team working between ophthalmologists and neurologists. However, there is a need for additional clinical trials to optimize management.

## Glossary

CSCR: central serous chorioretinopathy
EAE: experimental autoimmune encephalitis
EAU: experimental autoimmune uveitis
HLA: human leukocyte antigen
IU: intermediate uveitis
MBP: myelin basic protein
MMF: mycophenolate mofetil
OCT: optical coherence tomography
RRMS: relapsing-remitting MS
Th1: T-helper 1
TNF: tumor necrosis factor

## References

[1] Thorne JE, Suhler E, Skup M, et al Prevalence of noninfectious uveitis in the United States: a claims-based analysis. JAMA Ophthalmol 2016;134:1237–1245. 27608193 10.1001/jamaophthalmol.2016.3229

[2] Standardization of uveitis nomenclature for reporting clinical data. Results of the first international workshop. Am J Ophthalmol 2005;140:509–516. 16196117 10.1016/j.ajo.2005.03.057 PMC8935739

[3] Tsirouki T, Dastiridou A, Symeonidis C, et al A focus on the epidemiology of uveitis. Ocul Immunol Inflamm 2018;26:2–16. 27467180 10.1080/09273948.2016.1196713

[4] Olsen TG, Frederiksen J The association between multiple sclerosis and uveitis. Surv Ophthalmol 2017;62:89–95. 27491475 10.1016/j.survophthal.2016.07.002

[5] Thompson AJ, Baranzini SE, Geurts J, Hemmer B, Ciccarelli O Multiple sclerosis. Lancet 2018;391:1622–1636. 29576504 10.1016/S0140-6736(18)30481-1

[6] Forrester JV, Kuffova L, Dick AD Autoimmunity, autoinflammation, and infection in uveitis. Am J Ophthalmol 2018;189:77–85. 29505775 10.1016/j.ajo.2018.02.019

[7] Olsson T, Barcellos LF, Alfredsson L Interactions between genetic, lifestyle and environmental risk factors for multiple sclerosis. Nat Rev Neurol 2017;13:25–36. 27934854 10.1038/nrneurol.2016.187

[8] Burton A Multiple sclerosis: what's it got to do with your guts? Lancet Neurol 2018;17:207–208. 29274680 10.1016/S1474-4422(17)30468-4

[9] Lindner E, Weger M, Steinwender G, et al IL2RA gene polymorphism rs2104286 A>G seen in multiple sclerosis is associated with intermediate uveitis: possible parallel pathways? Invest Ophthalmol Vis Sci 2011;52:8295–8299. 21911588 10.1167/iovs.11-8163

[10] Wang Q, Su G, Tan X, et al UVEOGENE: an SNP database for investigations on genetic factors associated with uveitis and their relationship with other systemic autoimmune diseases. Hum Mutat 2019;40:258–266. 30614601 10.1002/humu.23702 PMC6590147

[11] de-la-Torre A, Silva-Aldana CT, Muñoz-Ortiz J, et al Uveitis and multiple sclerosis: potential common causal mutations. Mol Neurobiol 2019;56:8008–8017. 31161422 10.1007/s12035-019-1630-2 PMC6834745

[12] de Vos AF, Dick AD, Klooster J, Broersma L, McMenamin PG, Kijlstra A Analysis of the cellular infiltrate in the iris during experimental autoimmune encephalomyelitis. Invest Ophthalmol Vis Sci 2000;41:3001–3010. 10967057

[13] Kojima K, Berger T, Lassmann H, et al Experimental autoimmune panencephalitis and uveoretinitis transferred to the Lewis rat by T lymphocytes specific for the S100 beta molecule, a calcium binding protein of astroglia. J Exp Med 1994;180:817–829. 7520474 10.1084/jem.180.3.817 PMC2191664

[14] Adamus G, Amundson D, Vainiene M, et al Myelin basic protein specific T-helper cells induce experimental anterior uveitis. J Neurosci Res 1996;44:513–518. 8794942 10.1002/(SICI)1097-4547(19960615)44:6<513::AID-JNR1>3.0.CO;2-E

[15] Forooghian F, Cheung RK, Smith WC, O'Connor P, Dosch HM Enolase and arrestin are novel nonmyelin autoantigens in multiple sclerosis. J Clin Immunol 2007;27:388–396. 17436063 10.1007/s10875-007-9091-1 PMC2705966

[16] Keino H, Horie S, Sugita S Immune privilege and eye-derived T-regulatory cells. J Immunol Res 2018;2018:1679197. 29888291 10.1155/2018/1679197 PMC5985108

[17] Dendrou CA, Fugger L, Friese MA Immunopathology of multiple sclerosis. Nat Rev Immunol 2015;15:545. 26250739 10.1038/nri3871

[18] Baecher-Allan C, Kaskow BJ, Weiner HL Multiple sclerosis: mechanisms and immunotherapy. Neuron 2018;97:742–768. 29470968 10.1016/j.neuron.2018.01.021

[19] Forrester JV, McMenamin PG, Dando SJ CNS infection and immune privilege. Nat Rev Neurosci 2018;19:655–671. 30310148 10.1038/s41583-018-0070-8

[20] Kaskow BJ, Baecher-Allan C Effector T cells in multiple sclerosis. Cold Spring Harbor Perspect Med 2018;8:a029025. 10.1101/cshperspect.a029025 PMC5880159 29358315

[21] Baranzini SE, Jeong MC, Butunoi C, Murray RS, Bernard CC, Oksenberg JR B cell repertoire diversity and clonal expansion in multiple sclerosis brain lesions. J Immunol 1999;163:5133–5144. 10528220

[22] Van Kaer L, Postoak JL, Wang C, Yang G, Wu L Innate, innate-like and adaptive lymphocytes in the pathogenesis of MS and EAE. Cell Mol Immunol 2019;16:531–539. 30874627 10.1038/s41423-019-0221-5 PMC6804597

[23] Smith JR, Stempel AJ, Bharadwaj A, Appukuttan B Involvement of B cells in non-infectious uveitis. Clin Transl Immunol 2016;5:e63. 10.1038/cti.2016.2 PMC4771944 26962453

[24] Epps SJ, Coplin N, Luthert PJ, Dick AD, Coupland SE, Nicholson LB Features of ectopic lymphoid-like structures in human uveitis. Exp Eye Res 2020;191:107901. 31877281 10.1016/j.exer.2019.107901 PMC7029346

[25] Petrushkin H, Kidd D, Pavesio C Intermediate uveitis and multiple sclerosis: to scan or not to scan. Br J Ophthalmol 2015;99:1591–1593. 26338960 10.1136/bjophthalmol-2015-307269

[26] Frohman EM, Frohman TC, Zee DS, McColl R, Galetta S The neuro-ophthalmology of multiple sclerosis. Lancet Neurol 2005;4:111–121. 15664543 10.1016/S1474-4422(05)00992-0

[27] Guly CM, Forrester JV Investigation and management of uveitis. BMJ 2010;341:c4976. 20943722 10.1136/bmj.c4976

[28] Kempen JH, Van Natta ML, Altaweel MM, et al Factors predicting visual acuity outcome in intermediate, posterior, and panuveitis: the multicenter uveitis steroid treatment (MUST) trial. Am J Ophthalmol 2015;160:1133–1141.e9. 26386159 10.1016/j.ajo.2015.09.017 PMC4657141

[29] Ness T, Boehringer D, Heinzelmann S Intermediate uveitis: pattern of etiology, complications, treatment and outcome in a tertiary academic center. Orphanet J Rare Dis 2017;12:81. 28449695 10.1186/s13023-017-0638-9 PMC5408401

[30] Schmidt S, Wessels L, Augustin A, Klockgether T Patients with multiple sclerosis and concomitant uveitis/periphlebitis retinae are not distinct from those without intraocular inflammation. J Neurol Sci 2001;187:49–53. 11440744 10.1016/s0022-510x(01)00520-2

[31] Gordon-Lipkin E, Chodkowski B, Reich D, et al Retinal nerve fiber layer is associated with brain atrophy in multiple sclerosis. Neurology 2007;69:1603–1609. 17938370 10.1212/01.wnl.0000295995.46586.ae

[32] Lim LL, Silva DG, Lo TC, Pimentel RS, Butzkueven H, Hall AJ Uveitis in patients with multiple sclerosis in clinical trials of fingolimod: incidence, prevalence, and impact on disease course. Ophthalmology 2019;126:438–444. 30315901 10.1016/j.ophtha.2018.10.013

[33] Heath G, Airody A, Gale RP The ocular manifestations of drugs used to treat multiple sclerosis. Drugs 2017;77:303–311. 28181178 10.1007/s40265-017-0692-6

[34] Kempen JH, Kempen JH, Altaweel MM, et al Benefits of systemic anti-inflammatory therapy versus fluocinolone acetonide intraocular implant for intermediate uveitis, posterior uveitis, and panuveitis: fifty-four-month results of the multicenter uveitis steroid treatment (MUST) trial and follow-up study. Ophthalmology 2015;122:1967–1975. 26298715 10.1016/j.ophtha.2015.06.042 PMC4581989

[35] Liew G, Quin G, Gillies M, Fraser-Bell S Central serous chorioretinopathy: a review of epidemiology and pathophysiology. Clin Exp Ophthalmol 2013;41:201–214. 22788735 10.1111/j.1442-9071.2012.02848.x

[36] Macaron G, Ontaneda D Diagnosis and management of progressive multiple sclerosis. Biomedicines 2019;7:56. 10.3390/biomedicines7030056 PMC6784028 31362384

[37] Kappos L, Bar-Or A, Cree BAC, et al Siponimod versus placebo in secondary progressive multiple sclerosis (EXPAND): a double-blind, randomised, phase 3 study. Lancet (London, England) 2018;391:1263–1273. 10.1016/S0140-6736(18)30475-6 29576505

[38] Dick AD, Rosenbaum JT, Al-Dhibi HA, et al Guidance on noncorticosteroid systemic immunomodulatory therapy in noninfectious uveitis: fundamentals of care for UveitiS (FOCUS) initiative. Ophthalmology 2018;125:757–773. 29310963 10.1016/j.ophtha.2017.11.017

[39] Velazquez-Villoria D, Macia-Badia C, Segura-Garcia A, et al Efficacy of immunomodulatory therapy with interferon-beta or glatiramer acetate on multiple sclerosis-associated uveitis. Arch Soc Esp Oftalmol 2017;92:273–279. 28188020 10.1016/j.oftal.2016.11.018

[40] Hedayatfar A, Falavarjani KG, Soheilian M, et al Mycophenolate mofetil for the treatment of multiple sclerosis-associated uveitis. Ocul Immunol Inflamm 2017;25:308–314. 27379567 10.1080/09273948.2016.1178302

[41] Copland DA, Liu J, Schewitz-Bowers LP, et al Therapeutic dosing of fingolimod (FTY720) prevents cell infiltration, rapidly suppresses ocular inflammation, and maintains the blood-ocular barrier. Am J Pathol 2012;180:672–681. 22119714 10.1016/j.ajpath.2011.10.008 PMC3796282

[42] Lopalco G, Fabiani C, Sota J, et al IL-6 blockade in the management of non-infectious uveitis. Clin Rheumatol 2017;36:1459–1469. 28528519 10.1007/s10067-017-3672-z

[43] Brod SA, Bauer VL Ingested (oral) tocilizumab inhibits EAE. Cytokine 2014;68:86–93. 24845797 10.1016/j.cyto.2014.04.003

[44] Ringelstein M, Ayzenberg I, Harmel J, et al Long-term therapy with interleukin 6 receptor blockade in highly active neuromyelitis optica spectrum disorder. JAMA Neurol 2015;72:756–763. 25985228 10.1001/jamaneurol.2015.0533

[45] Roemer S, Bissig A, Rocca A, Du Pasquier R, Guex-Crosier Y Efficacy of natalizumab in intermediate uveitis related to multiple sclerosis: a case report. Klin Monbl Augenheilkd 2018;235:476–477. 29452449 10.1055/s-0043-124756

[46] Dick AD, Meyer P, James T, et al Campath-1H therapy in refractory ocular inflammatory disease. Br J Ophthalmol 2000;84:107–109. 10611109 10.1136/bjo.84.1.107 PMC1723242

[47] Miserocchi E, Modorati G Rituximab for noninfectious uveitis. Dev Ophthalmol 2012;51:98–109. 22517208 10.1159/000336188

[48] Mandal P, Gupta A, Fusi-Rubiano W, Keane PA, Yang Y Fingolimod: therapeutic mechanisms and ocular adverse effects. Eye (Lond) 2017;31:232. 27886183 10.1038/eye.2016.258 PMC5306460

[49] TNF neutralization in MS: results of a randomized, placebo-controlled multicenter study. The lenercept multiple sclerosis study group and the University of British Columbia MS/MRI Analysis Group. Neurology 1999;53:457–465. 10449104

[50] Beauchemin P, Carruthers R MS arising during tocilizumab therapy for rheumatoid arthritis. Mult Scler 2016;22:254–256. 26743640 10.1177/1352458515623862

[r-51] Additional references e1-e74 available at: http://links.lww.com/NXI/A332.

